# New Chemical and Philosophical Apparatus

**Published:** 1855-10

**Authors:** Alexander Means

**Affiliations:** Oxford, Georgia


ARTICLE VI.—*New Chemical and Philosophical Apparatus.* By ALEXANDER MEANS, M. D., Oxford, Georgia.

At the recent Fair of the Southern Central Agricultural Association, held in Atlanta, I was induced to furnish for exhibition, some specimens of new chemical and philosophical instruments, projected by myself, and manufactured according to order, by the well known and skillful philosophical instrument makers, Chamberlain & Ritchie, Boston, Massachusetts. The pieces of apparatus presented, however, were never *intended* for public inspection, but were prepared with special reference to the wants of my own Laboratory and Lecture Room.

In accordance with the wishes of those in whom I have confidence, but at very short notice, I have consented to furnish the "Journal" with a description of the articles exhibited, together with the *uses* to which they were designed severally to be appropriated.

For about 20 years past, I have been engaged in public and private manipulations with an extensive supply of the most approved instruments in general use during that time, but have found, in some instances, no form of apparatus which would satisfactorily illustrate, to the eye of a college class, or of a popular audience, certain principles or laws, which I desired attractively and forcibly to present. In other cases, I occasionally found great inconvenience from the defective construction of the instrument employed, and I was therefore, induced to tax my own resources for the supply of those deficiences.

The three instruments, therefore, to the explanation of which I have been requested to devote the present article, have been the result of experience and reflection; and may, I trust, serve to facilitate some of the processes of the Laboratory, and aid in illustrating some sublime laws of mechanical philosophy, connected with stellar and planetary motion. The author distrusts himself too much to present any claims to extraordinary merit for this small contribution to science, and while he properly appreciates the favorable decision made by the examining committee, and gratefully accepts their generous award,* he is inclined to attribute *both*, as much to the laudable spirit which actuated the Association, to encourage future scientific improvements and inventions in this novel department of our State Fair, as to any intrinsic claims of the instruments themselves.

Each article will be separately considered, and dispatched with as much brevity as may consist with a clear apprehension of its construction and use. And even this, I fear, may be regarded as tediously long.

1ST.—A DILATATION STAND, *for illustrating the expansion of different fluids under the same increments of heat.*

This instrument was designed to furnish a popular exhibition of the law of expansion in fluids, rather than the means of *accurately* estimating the amount of that expansion. For close inspection and nice results, the syphonic glass-tube, of varying calibre, employed by Dulong and Petit, is not surpassed, or equalled, by any instrument in use. This, however, will only allow of the examination of one liquid at a time. It is, for many practical purposes, exceedingly desirable to know the proportional dilatation of different liquids—water and mercury especially, among the number.

It is proper to remark, that the *apparent* expansion of fluids in glass tubes, on the application of heat, is less than the *absolute* expansion, as the dimensions of containing vessel, are at the same time enlarged. This, in rising from 32° to 212° Far-

*A beautifully embossed silver pitcher, valued at $25 00.

enheit, or from the freezing to the boiling point of water. Mercury exhibits an *apparent* expansion of $\frac{1}{64.8}$ of its volume, or 64.8 measures, become 65.8, while its *real* expansion is $\frac{1}{55.5}$ or, 55.5 measures become 56.5. Again, spirits of wine expands $\frac{1}{9}$ of its entire volume, or 6 times as much as mercury; so that some alcoholic liquors will measure 5 per cent. more during the heat of summer than during the cold of winter. To make the action of this law of easy comprehension, the Dilatation Stand has been constructed.

It consists of a heavy circular pedestal of iron, neatly finished and painted; into the center of which, is fixed an upright shaft of the same metal, $\frac{1}{4}$ of an inch in diameter, and 18 inches high, surmounted by a small stationary brass ring, through which the finger may readily pass. A small *ball* of iron —perforated for the passage of the shaft, and from which project laterally, and at right angles with each other, 4 little rods of iron 2 inches long, and terminated by rings $1\frac{1}{2}$ inches in diameter, and lying horizontally—slides down the shaft to the pedestal, if necessary, where it may be fixed by a thumb-screw. Another similar ball with crucial rods, and smaller rings, is attached by a thumb-screw above. Into this frame are introduced 4 small, well blown bolt-heads of transparent glass, with stems of uniform calibre, and all as nearly as possible of the same shape and dimensions. The ball being about $1\frac{1}{2}$ inches in diameter, and the neck or stem $\frac{1}{3}$ of an inch, and 18 inches long. The balls are allowed to rest on the lower rings, which to secure safety, are wrapped with cotton-wadding—while the stems pass through the smaller rings above, and are steadied by soft, split corks. Into these balls the several fluids to be tested, should be introduced, which is best done by a long-tubed, small funnel, with capillary orifice. The fluid should ascend in each stem precisely to the same height, and its position be marked by a colored thread. Thus arranged, the entire system is let down by the aid of the brass-top ring, into a vessel containing water at any temperature from 32° to 212° Farenheit, and immersed all together to the same depth. The difference in the elevation of the several columns, will indicate the difference in the expansibilities of the fluids employed.

2ND.—THE TUBULAR LAMP-STAND—

This instrument, as improved, consists of a heavy iron pedestal, of genteel finish, about 10 inches long and 4 wide, rounded at each extremity; from the center of which rises a perpendicular shaft of iron, ¼ of an inch in diameter, and 20 inches high. Over this, slips easily a brass tube about 5 inches long, with a thumb-screw at the lower extremity, by which to attach it at any point, to the upright iron shaft. When this is let down to the foot of the stand, *another* tube 15 inches long, with thumb-screw, as the former, is slipt over the iron shaft and is attached at any point above the preceeding one. Over these tubes slide the rings, which in one form of the Lamp-Stand are *double*, and consist of five pairs of assorted sizes; and in another form, single. Both forms are now manufactured in Boston, by Mr. E. S. Ritchie, No. 313 Washington St. The principal advantages of this form of stand over others in common use, may be enumerated as follows:

1. Two Florence Flasks may be kept over lamps at the same time, which is sometimes desirable.

2. Two such Flasks with a re-curved glass tube in each may be connected with a *third* vessel, supported on a supplementary *side-ring*, as a common receiving matrass—all on the same stand—their distances, apart when once arranged, remaining unchanged, and the entire system, with lamp burning, readily transferable to any part of the room.

The convenience of this latter arrangement, may be beautifully illustrated by heating to ebullition Aqua Ammonia in the one Flask, and Hydrochloric Acid in the other; and then, by the bent glass tubes, uniting their vapors in the *third* vessel where the Hydrochlorate of Ammonia is instantly found, and deposited in snow-white powder in the interior.

3. A Flask once fixed closely between two rings may be raised to any desired height above the lamp-flame, by sliding the tube with rings and flask attached, up the iron shaft.

4. The Stand may be increased, if necessary, for side-table use, to almost *twice* its ordinary height.

5. The whole upper tubes and rings may be removed, if desirable, so as to use more freely a large porcelain evaporating dish on the large lower ring.

6. The double *iron pedestal* gives *firmness* and *security* to the whole, and allows the use of a Portable Furnace upon it without danger of burning it.

7. Long wooden thumb-pieces, terminated by iron screws, are substituted for the old metallic short screws, or buttons, which almost invariably burnt the fingers of an operator, when the flask, rings and shaft, had been for a few minutes subjected to the ordinary heat of a Spirit Lamp.

3RD.—GLASS GLOBE, AXIS AND STAND, *for Planetary Organization and Revolution on La Place's Theory.*

The construction of this instrument was suggested by the elementary experiment, reported by Professor Plateau of Ghent, a few years since, who used a simple *box* of glass, with axle, disk, &c. Our Apparatus consists of a *large glass globe*, about 10 inches in diameter, with thick walls and well blown, mounted with brass socket and stop-cock upon a large, circular, mahogany stand, which should be lead-weighted underneath. Above, is a short neck and orifice 2½ inches wide, with ground surface, to be used, if desired, on the Air Pump, or with glass or brass plates. When used for *astronomical* illustrations, it is surmounted by a brass cap which fits on the neck closely, and has a small orifice in the center, through which passes a vertical metallic *shaft*, that rests with sharp point upon a little indentation or socket at the bottom of the globe—the upper end of this axis, terminating in a small crank, to allow of revolution by the fingers. A little below the center, is a small *disk*, ⅜ of an inch in diameter, through which the axis passes, and in which it fits tightly.

For experiment, the globe is filled at least two-thirds full of alcohol, reduced by water so as precisely to suit the specific gravity of the *oil* to be used. When thus prepared, the oil should be poured in while the cap and axis are removed. I find thick *Castor Oil* to be best suited for our purpose, and if highly colored by age or accident the better, because the more distinctly seen. The earth's attraction being thus neutralized, the oil introduced will instantly assume a perfectly globular shape in the centre of the fluid. A quantity equal in size to a blue plum is altogether sufficient.

The cap should now be applied, and the axis carefully let down, and by gentle management the dish made to occupy the centre of the oil-globule, when gradual revolution of the axis should commence, and then all the phenomina, illustrative of the philosophical principles hereafter enunciated, may be witnessed.

1. The molecules of all masses, when left free to move upon each other, seek to approach a centre common to the whole. When all remote or secondary attractions are destroyed or removed, therefore, a perfectly globular form must be the result. We see an attempt at conformity to this law in the form of the dew-drop, globules of fallen mercury, &c., only flattened below by the earth's attraction.

2. All such bodies, revolving upon an axis, will assume the form of an *oblate spheroid*—being flattened at the poles and extended at the equator.

3. If the motion be increased so that the *centrifugal* becomes greater than the *centripetal* or attractive force, the equatorial portions, which pass through a greater space, in the same time, and whose velocity is therefore greater, will be thrown off from the mass.

4. If at the time of detachment all portions of it are equally distant from the common axis, it will be simultaneously separated as an unbroken ring, beautifully simulating the rings of Saturn, which encircle that gorgeous planet.

5. If unequally distant from the axis at the time of separation—as is commonly the case—the detached portion will be broken up into fragments, each of which will conform to its own center—making smaller globes, which will at once commence revolution around the original mass from which they were ejected.

6. They will all conform in their line of revolution to that of the parent mass, and all their orbits will be found nearly parallel with the plane of the equator of that revolving mass, as admirably exemplified on a magnificent scale in the revolution of satellites around their Primaries, and Primaries around the sun in our solar system.

In conclusion of this wearisome detail, I may be permitted to add that the globe used in the last-named set of experiments is also used for some of the most brilliant exhibitions in Pneumatics and Electricity—as the combustion of Phosphorus in Oxygen gas, the Aurora Borealis, &c.

These instruments have been adopted by Mr. E. S. RITCHIE, Philosophical Instrument maker, Boston, and are furnished to order.

